# AI and Big Data in Healthcare: Towards a More Comprehensive Research Framework for Multimorbidity

**DOI:** 10.3390/jcm10040766

**Published:** 2021-02-14

**Authors:** Ljiljana Trtica Majnarić, František Babič, Shane O’Sullivan, Andreas Holzinger

**Affiliations:** 1 Department of Internal Medicine, Family Medicine and the History of Medicine, Faculty of Medicine, University Josip Juraj Strossmayer, 31000 Osijek, Croatia; ljiljana.majnaric@mefos.hr; 2 Department of Public Health, Faculty of Dental Medicine, University Josip Juraj Strossmayer, 31000 Osijek, Croatia; 3 Department of Cybernetics and Artificial Intelligence, Faculty of Electrical Engineering and Informatics, Technical University of Košice, 066 01 Košice, Slovakia; 4 Department of Pathology, Faculdade de Medicina, Universidade de São Paulo, 05508-220 São Paulo, Brazil; doctorshaneosullivan@gmail.com; 5 Institute for Medical Informatics, Statistics and Documentation, Medical University of Graz, 8036 Graz, Austria; andreas.holzinger@medunigraz.at

**Keywords:** multimorbidity, artificial intelligence, machine learning, population aging, chronic diseases

## Abstract

Multimorbidity refers to the coexistence of two or more chronic diseases in one person. Therefore, patients with multimorbidity have multiple and special care needs. However, in practice it is difficult to meet these needs because the organizational processes of current healthcare systems tend to be tailored to a single disease. To improve clinical decision making and patient care in multimorbidity, a radical change in the problem-solving approach to medical research and treatment is needed. In addition to the traditional reductionist approach, we propose interactive research supported by artificial intelligence (AI) and advanced big data analytics. Such research approach, when applied to data routinely collected in healthcare settings, provides an integrated platform for research tasks related to multimorbidity. This may include, for example, prediction, correlation, and classification problems based on multiple interaction factors. However, to realize the idea of this paradigm shift in multimorbidity research, the optimization, standardization, and most importantly, the integration of electronic health data into a common national and international research infrastructure is needed. Ultimately, there is a need for the integration and implementation of efficient AI approaches, particularly deep learning, into clinical routine directly within the workflows of the medical professionals.

## 1. Introduction

Societies in industrialized countries worldwide are facing an increasing burden of chronic diseases, including type 2 diabetes, cardiovascular and neurodegenerative diseases, and various cancers. This negative trend is the result of an aging population and the prevalence of “modern” lifestyles, such as the consumption of industrially processed foods, predominantly sedentary work, and increasing chronic psychological stress, which are known to accelerate aging and the development of age-related diseases [1,2].

Chronic diseases in the same person rarely appear as a single disease; instead, two or more diseases coexist, which is called multimorbidity [3]. Patients with multimorbidity raise a concern of both policymakers and healthcare providers because of the complex care needs, which requires various healthcare providers and services to deliver care for these patients [4]. The general practitioners (GPs) are faced with the demanding task of integrating different recommendations and prescriptions of these multiple providers [5].

Moreover, current clinical guidelines are disease-oriented, further complicating decision making for these patients [6]. Even recommendations for managing single diseases for these patients may be uncertain because patients with multimorbidity are usually excluded from clinical trials. The delivery of care and the patient self-management may be constrained by complicated medication regimens and information burden [5]. Recommendations that are given to a patient for several single diseases may be mutually conflicting and produce harm rather than good [7].

Multimorbidity has been shown to have a significant negative impact on patient outcomes, and not all patients with multimorbidity have the same risk for adverse outcomes. It has been shown to depend on the number of comorbidities, but also on certain combinations of diseases that a person has [8]. Some disease combinations occur randomly, as some diseases are very common in the population, such as hypertension, while other diseases tend to accumulate [9]. Disease clustering is usually based on common pathophysiology, as evident from the common appearance of cardio-metabolic and vascular disorders, although in some cases, causes are less clear [10]. However, the classical methods of measuring multimorbidity that have been used in epidemiologic surveys and are based on counting diseases are not adequate to capture reliable pre-existing conditions. [11,12].

There is no knowledge base to adequately address multimorbidity problems in terms of patient-centered solutions in predicting specific outcomes and determining personalized treatments [13,14]. This is due to the fact, that no methodological framework has been developed that adequately manages the complexity of multimorbidity. To clarify what we mean by complexity, we illustrate it in the following examples.

Typically, in an older population (>60), when the number of chronic diseases increases, the prevalence of mental disorders, such as anxiety and depression, also increases [15,16]. Somatic and mental disorders are not wholly distinct, as these two share some common mechanisms, such as in associations of mental disorders with cardio-metabolic and chronic pain conditions [17,18,19]. The significance of these findings lies in the demonstrated adverse effects of mental disorders on the course of chronic somatic diseases and on health outcomes, which may justify actively seeking these disorders in older, multimorbid patients in primary care settings [20,21,22].

Another feature of aging and multimorbidity is the presence of health conditions beyond the traditional diagnostic label that can negatively affect the quality of life and functional abilities of older people [23,24]. Of these conditions, which include disorders including walking difficulties, vision and hearing loss, impaired balance, dizziness, predispositions for falls, incontinence, chronic pain, and delirium, cognitive impairment and frailty, have attracted the most attention of researchers due to the proven impact of these disorders on important adverse outcomes, such as dementia, disabilities, and ultimately death [23,25]. Frailty is defined as the state of decreased homeostatic reserves in multiple physiologic systems, presented with symptoms of shrinking, slowness, and weakness, and can be considered as the final common pathway in the development of multimorbidity [25,26]. Cognitive impairment is a highly prevalent disorder in the elderly, and its progression towards dementia is increased in persons with cardiovascular disorders, especially when there is comorbidity with depression [27]. The disabling effect on health is highest in cases where there is a coexistence of frailty with cognitive impairment [28].

The examples described above illustrate the complex relationships that exist in older people between somatic illnesses and psychological, cognitive, and functional impairments. The degree of complexity is even greater when the effects of treatments are included in the consideration of multimorbidity. In addition to beneficial effects, pharmacologic treatment, especially when given for multiple indications, can also be counterproductive because of unpredictable drug-disease interactions. Indeed, many symptoms and functional limitations in the elderly are the result of pharmacological treatments [24].

Understanding this complexity proves challenging, as the study of patients with multimorbidity must go beyond the scope of well-defined categories, such as disease labels. Because classical statistical methods cannot provide an adequate framework for stratifying these patients, there is a need for a more comprehensive research framework [14].

One possible solution seems to lie in the AI approaches of machine learning (ML) and Big Data (BD) technologies, which have already provided fruitful results in solving complex problems in many other areas of human activity, such as industry, finance, and marketing [29,30].

The purpose of this review is to summarize the limitations of current approaches to data analysis and to present the potential and shortcomings of alternative methods in multimorbidity research. The recently published paper by Hassaine et al. provided an in-depth analysis of methodological advances in identifying multimorbidity-associated patterns hidden in data sequences in electronic health records (eHRs) and methods for tracking the time courses of these patterns [31]. On the contrary, in fact, because our overview shows what the problem of multimorbidity research looks like in the eyes of medical professionals.

Consequently, this paper aims to increase the understanding of medical laypersons of the essential ML/BD research approaches and provides some tips on how to overcome barriers to broader implementation of these methods in multimorbidity research, with the ultimate goal of improving the quality of medical practice. The authors reflect on these issues based on their work experiences and emphasize the need for closer collaboration between medical experts and data scientists throughout the complex problem-solving process, from problem definition to data and method selection to interpretation of research results.

## 2. The Time for the Paradigm Change in Research on Multimorbidity

In the classical research approach of the medical field, the scope of research questions is limited to those for which answers can be provided within the set of well-defined statistical methods. Data analysis is driven by the well-defined hypothesis to be proven or rejected (a hypothesis-driven approach). The prerequisite for this approach is a well-documented knowledge base and a data set collected according to strict protocols [32].

The multiple regression models (MLR) of classical statistics, as essential tools for making predictions, are methods per se, i.e., the structure of the model is predetermined and remains fixed during the modeling process [30]. The MLR models are based on the assumptions of the independence of the input variables, linearity between dependent and independent variables, normality of the residuals (proposing the balanced data distribution), and the absence of endogenous (confounding) variables [33]. The strong rules on which the models rely, limit the scope of research questions and the types of data that can be used for analysis. For example, these models are not suitable for problems that do not fit the linear models or utilize a large number of variables [34]. Only classical statistical methods are not enough to address multimorbidity questions, where components are mutually interrelated within the complex network. So, the components’ emerging properties, not only the component number and the structure, maybe a critical determinant of the outcome.

Suppose we want the theoretical paradigm shift in research on multimorbidity to reach the level of a practical implementation. In this case, we need to change the approach to problem solving in medical research [35].

In addition to the classical, reductionist approach, which dominates scientific reasoning today, the approach from the aspect of the complex systems should be used as a complementary one when studying phenomena associated with chronic diseases and multimorbidity.

The scientific reasoning that relies on the paradigm of reductionism states that the cause-effect relationships in the real world can be described by a limited set of logical rules and static mathematical models [36]. This concept assumes that the system, to be understood, should be broken into its components, and then analysed. Scientific reasoning relies on durable logic and clear hypotheses, which excludes contradictions or uncertainties.

However, biological systems behave like complex systems [37]. In a complex system, its property emerges through interactions of its components. The distinct phenomena that arise from these interactions include: spontaneous order (self-organisation), non-linear relationships (change in one entity does not correspond with constant change in the other entity), redundancy (the existence of several complementary pathways), feedback loops (a chain of cause-and-effect that makes not possible a conclusion on the causal relationships), and a high level of adaptability (functionality). It is always important to note that many diverse modalities contribute to a decision [38].

Based on research in molecular science and epidemiological observations, the evidence is growing to allow for an integrated view on aging and the development of chronic diseases and multimorbidity. This process can be represented by a range of trajectories that differ in dynamics of chronic diseases and functional impairment accumulation over time [39]. The person’s position at the trajectory is influenced by the interplay of inner and outer factors, including genetics, environmental, social, and lifestyle factors, and the dynamics of their change over time.

This view can be placed within the concept of complexity in biological systems, according to which aging is the process of progressive disruption in the multiple communication channels, which otherwise connect organs, control systems, and regulatory loops, allowing for information to flow between them [40]. The disruption in these communication channels is associated with a decline in the body’s functional capabilities and the dispersion of phenotypes, in the form of the appearance of aging diseases, disabilities, and frailty [36,40]. Although being heterogeneous, older people are yet significantly like each other due to overlapping disorders.

The adoption of scientific reasoning from the aspect of the complex systems is expected to improve our ability to make conclusions on phenomena associated with multimorbidity, despite the lack of knowledge on relationships that exist between the components of the system. This type of thinking takes multiple elements into account when making conclusions and operates with the terms “chance/probability”, rather than “causality/determination” [36]. In searching for ways and methods for solving complex problems, pieces of different theories, mixed methodologies, and interdisciplinary approaches can be used together [36]. The choice of methods is a great part of the researcher’s responsibility and depends on his/her knowledge and intuition (and is, to some extent, subjective).

## 3. The Machine Learning/Big Data Approaches and Challenges in Research on Chronic Diseases and Multimorbidity

Due to the invention of new technologies in medicine and healthcare, in the last decades, such as digital imaging techniques and molecular biology diagnostics, and to the establishment of patient registries and electronic HRs in many countries in Europe and wider, there has been a rapid growth in data quantity and complexity, in both medical research and clinical practice. It made the classical research approach no more sufficient to meet the challenges of the data analysis. The methods and techniques from ML/BD of AI approaches have been emerging to provide alternative solutions [29,30] (Table 1).

This alternative research approach has on disposition a broad range of analytical tools which application is a part of the knowledge discovery (KD) multi-step protocol of data analysis, in which the step “pattern extraction in data” precedes the step “evaluation of the knowledge base”, this data analytical approach being called “a data-driven approach” [41,42].

Algorithms and tools from this research approach have been compiled from different analytical fields, including mathematics, statistics, and computer science, to let the computers analyze datasets that are large in volume, require a high-velocity analysis, and shows a high level of diversity and complexity (high-level dimensionality, complex relationships, and other complications) (Table 2) [41,42,43,44].

The ML/BD analytical approach enables challenging the paradigm shift in medical science and healthcare towards precision medicine [45].

The typical representation of the association rule is IF X THEN Y, where X, Y are subsets from a whole set of items [47]. ARM represents a popular mining method because of its easily interpretable results as a set of rules. TARs express that a set of items tends to appear along with another set of items in the same transactions, in a specific time frame [48].

LR is a method of modelling the probability of an outcome that can only have two values [49]. It aims to find the best fitting model describing the relationship between the dichotomous variable (dependent variable–it contains data coded as 1/0, TRUE/FALSE, yes/no, etc.) and asset of independent variables.

NB requires a small number of training data to estimate the parameters necessary for classification [50]. It uses the probabilities of each attribute belonging to each class to make a prediction. NB simplifies the calculation of probabilities by assuming that the probability of each attribute belonging to a given class value is independent of all other attributes. To make a prediction, it calculates the probabilities of the instance belonging to each class and selects the class value with the highest probability.

**Figure 1:**
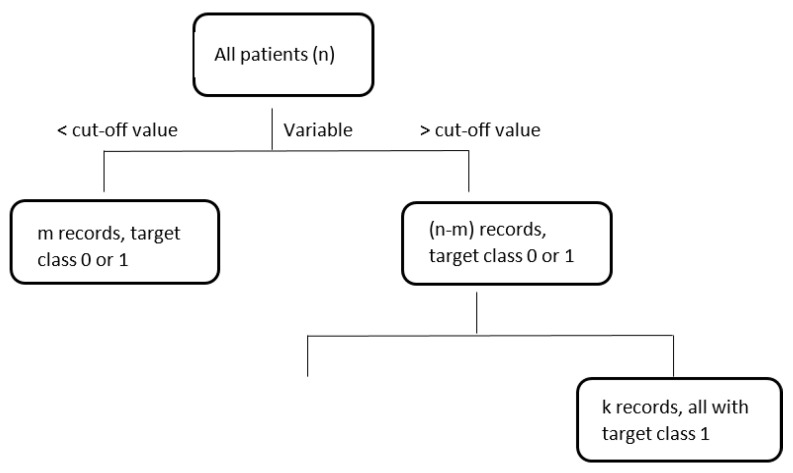
An example of decision tree.

DTs is a flowchart-like tree structure, where each non-leaf node represents a test on an attribute, each branch represents an outcome of the test, and leaf nodes represent target classes or class distributions, see Figure 1. Each DTs algorithm uses its own splitting criteria like information gain or Ginni-index to create branches and leaves [51]. In the case of the new record, this goes through the branches based on related conditions and finishes in the leaf node, after which no further branching is possible. And this leaf node determines a target class for the new record with relevant accuracy or other metrics.

RF model consists of a collection of tree-structured classifiers. It uses a bagging method, i.e., it contains the randomly sampled subsets of the training data, fitting a model to these smaller data sets, and aggregating the predictions [52]. An out-of-bag-error is used as an estimate of the generalization error.

**Figure 2:**
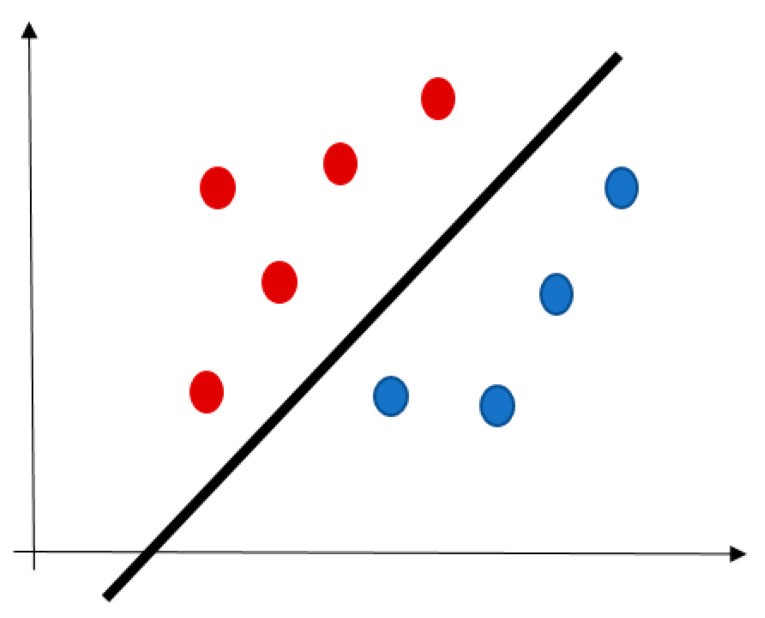
An example of SVM hyperplane (red and blue points are the training records).

SVM plots each record as a point in n-dimensional space (where n is a number of input variables) [53]. The value of each variable represents an ordinate. Then, SVM performs classification by finding a hyperplane distinguishing the two target classes very well, see Figure 2. This algorithm is not suitable for the larger datasets because of a long training time.

**Figure 3:**
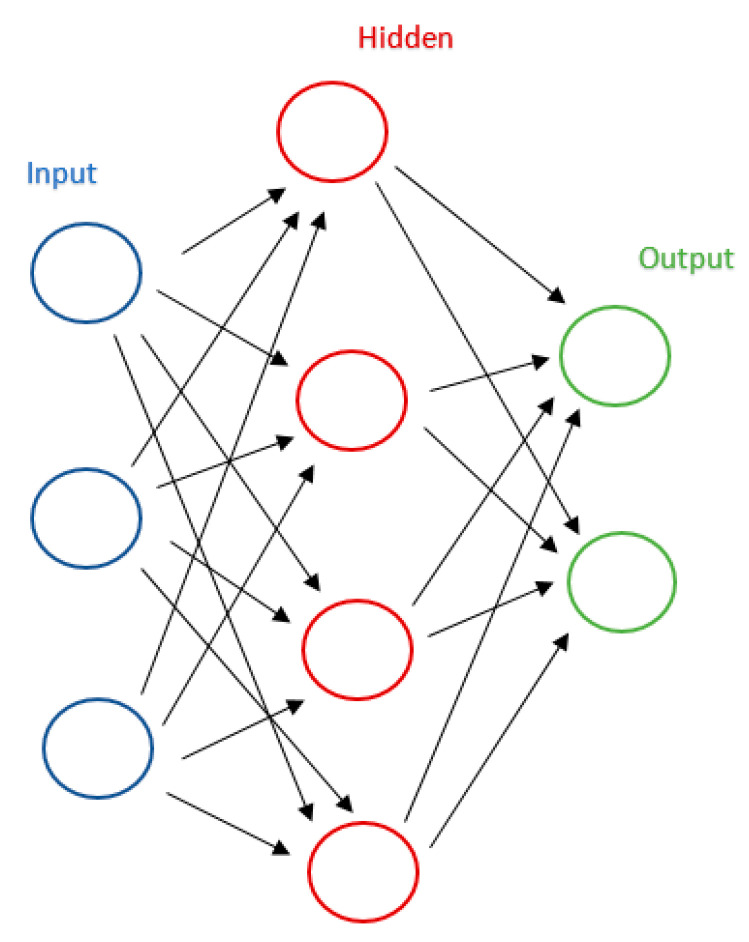
An example of three-layers neural networks.

Neural networks are a computational model inspired by biological neural networks, which is based on a large collection of simple connected units called artificial neurons, see Figure 3. We will use this method if we do not want to know the decision mechanism. They work like a black box, i.e., we know the model is some non-linear combination of some neurons, each of which is some non-linear combination of some other neurons, but it is near impossible to say what each neuron is doing [54]. This approach is opposite to the DT or SVM.

**Figure 4:**
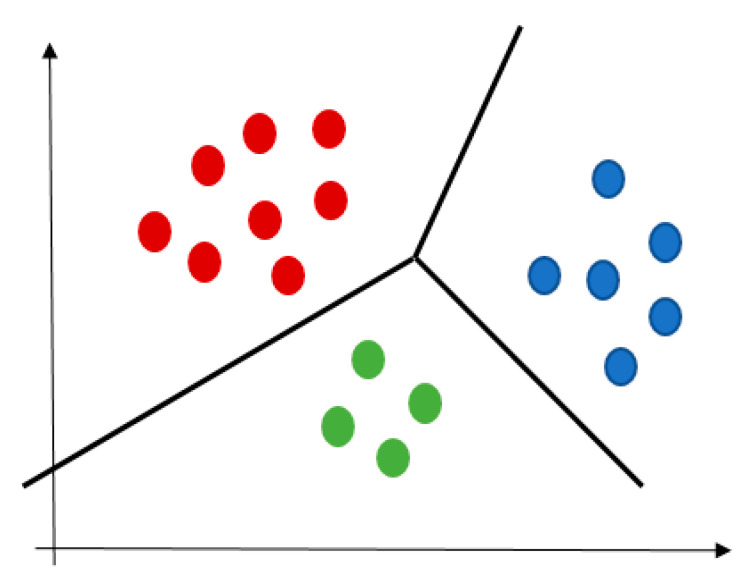
An example of clustering result, individual colors represent different clusters.

A cluster is a collection of data that describes objects like each other but dissimilar to objects in different clusters, see Figure 4. The K-mean algorithm aims to partition a set of objects into k clusters so that the resulting intra-cluster similarity is high and the inter-cluster similarity is low [55]. The intra-cluster similarity is measured with the mean value of distances between the objects in the cluster, which can be considered as the cluster’s centre.

**Figure 5:**
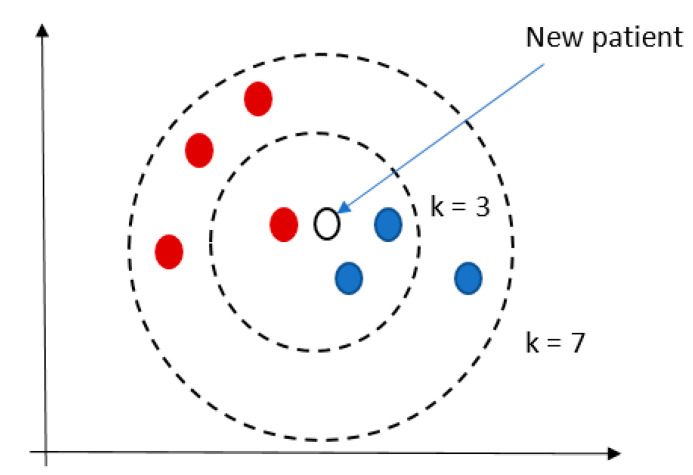
An example of KNN result.

KNN is commonly based on the Euclidean distance between a test sample and the specified training samples [56]. The output is a class membership, i.e., the record is classified by a majority vote of its neighbors, with the record being assigned to the class most common among its k-nearest neighbors, see Figure 5. A user specifies the parameter k.

PC analysis allows a visualization of the data set in order to reduce the dimensionality of multivariate data to two or three principal components with minimal loss of information [57]. These components correspond to a linear combination of the originals and represent the data’s maximum variance direction.

**Figure 6:**
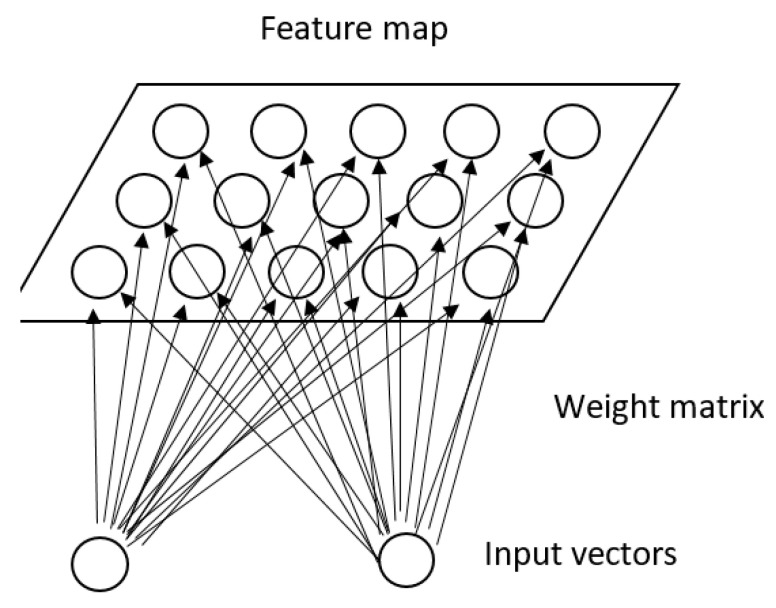
An example of SOM.

SOMs are representative of NN used for dimensionality reduction. This method is based on UNV learning to produce a low-dimensional, discretized representation of the training samples’ input space [58]. Its representation is called a map and uses a neighborhood function to preserve the topological properties of the input space, see Figure 6.

LCA is a statistical method for identifying unmeasured class membership among subjects using categorical and/or continuous observed variables [59].

Graph-based DM is typically applied on databases represented as graphs and mines topological substructures embedded in the data [60]. The goal is not only extracting the structure but identify potential interesting substructures.

NLP represents the automatic handling of natural human languages like speech or text that gives computers or robots the ability to understand the human languages, e.g., disease prediction based on the health records or patient’s speech [61].

NMF and its extension called NTF are emerging methods to decompose a nonnegative data matrix into a product of lower-rank nonnegative matrices or tensors (i.e., multiway arrays) [62]. The results are sparse and easily interpretable.

The algorithms of ML/BD techniques applied to the dataset must often learn about the data structure to obtain the model that is yielding the optimal description of the data, which makes them distinct from the rigorous MLR models of the classical statistics [32,33]. In addition, the ML/BD-AI research approach allows for a wider scope of research questions by allowing methods to adapt to the research question. It is likely to bring clinical studies into the real-world scenario, in which population heterogeneity is not an obstacle for performing research [29,43]. Yet, the critical point that makes the ML/BD-AI research approach favorable for studying multimorbidity, over the classical statistical methods is the ability of this approach to revealing the latent spaces and time trends in the data, irrespective of the data structure. The complex data structure in problems associated with multimorbidity arises from multiple and overlapping disorders and causing factors, external sociodemographic and internal biological, and poorly understood mechanisms that drive the dynamics between them (Table 3) [41,45,63].

A tendency of ML procedures for automatization is likely to diminish the role of a medical expert in the analytic process. However, it is not valid. This role is important because most medical domain problems are challenging and cannot be solved solely by applying the automated process of data analysis and without the medical expert’s guidance [64].

The majority of studies performed so far have faced some of the concepts of the ML/BD research approach, such as, i.e., the use of the large datasets to generate the alternative models (compared to those that are used in clinical practice), personalized adjustment of the guidelines’ recommendations, and the use of a combination of data sources to discover new biomarkers or to identify unknown mechanisms. These studies have yet to stay within the framework of the theory of reductionism, focusing on studying single diseases, whereas an integrated approach that on the contrary, would be more suitable to cope with the complexity of multimorbidity has stayed out of the scope. Many of these studies were shown useful in narrowing questions associated with multimorbidity and in creating new hypotheses, but they are not sufficient to answer the questions related to clustering of multiple diseases, which otherwise would be necessary to establish the efficient prevention and management strategies to cure people with multimorbidity.

Some of the questions that would be of interest for practicing doctors to be provided by the answers, include: which patients with multiple comorbidities are at risk for which specific outcomes, and which ones would benefit from which treatments; which risk factors and pathophysiology disorders refer to which patient groups, and which mechanisms are responsible for patient transition from one trajectory to another?

The most prevalent topics were those related to early diagnosis, personalized treatment, and prediction of outcomes of some chronic diseases, such as cancer, diabetes, and Alzheimer’s dementia. These diseases are known for their serious outcomes and are associated with many comorbidities so that by themselves, they are characterized with a high level of complexity. With the recent invention of high throughput (-omics) techniques in the medical domain that were thought to realize personalized patient care, the ML/BD analytical methods have received an additional stimulus for development.

In a review paper focusing on the current methods for cancer risk and recurrence prediction, Richtera and Khoshgoftaara discussed methodological concerns associated with the modelling process [65]. The authors emphasize the value of the structured data that is widely available in eHRs and national registries, such as clinical, social, and behavioral data, for modelling cancer prediction and prognosis at the population level. The molecular medicine data, such as data from genomics and proteomics, could be useful in determining personalized treatments but is not feasible for population screening. Techniques used in the models, either statistical method, are the cox proportional hazard regression model and the risk survival analysis, or ML methods, like SVM, ANNs, DTs, and RF. Most often, however, a combination of methods is used to achieve a valid prediction model. The data analyst should work closely with the domain expert in determining which type of models works best for which research problem.

In the paper specifically focused on using ML methods for cancer prediction and prognosis, Kourou et al. emphasized the ability of ML tools, like SVM, DTs, ANNs, and BNs, for detecting the key features from the large and yet unexplored input space in order to provide the accurate predictive models that can be used in everyday practice [46]. By the selection of new features used from an unknown input space, these models could enlarge the knowledge of factors that may influence cancer development and progression. This way, these models could fuel further research. A similar idea stayed behind the study of Rajan and Prakash [66]. These authors developed the ANN model for early diagnosis of lung cancer using the information on behavioral and social risk factors and symptoms, rather than on images and laboratory findings, making this model broadly accessible. This approach is valuable from the practical perspective, knowing that lung cancer is a leading cancer cause of death. There are no blood biomarkers or some simple methods that could be used for screening in the population.

Similar methodological issues burden the modelling procedures associated with the diagnosis and progression of diabetes type 2. In Zou et al., the emphasis is put on the importance of data pre-processing and reduction methods, and on the model generalization methods, as prerequisites for creating accurate ML models for diabetes diagnosis prediction [67]. Without the participation of a medical expert, who is able to define the research problem exactly define the research problem, there is a threat that the modelling procedure will serve merely as a confirmatory analysis of the applied ML method, without any detachment made from the existing knowledge. In the study of Sacchi et al., on the contrary, the problem was tackled on diabetes as a chronic disease, which progression over time is associated with accumulation of complications, and for which, the prediction is challenging to perform [68]. In this study, information was used on changes over time in medication prescription rates, together with a time series analysis, to the model prediction of chronic diabetic complications. How prediction of diabetes complications can be difficult when numerous variables interact in a complex and non-linear manner, and throughout the disease process, it was illustrated in the study of Yousefi L, et al. [69]. An intriguing issue is how to cope with the complex biological structure and dynamics of change over time. To solving this task, it would require complicated methods and modelling procedures. The authors stated that the best way to cope with this complexity is by dividing patients into smaller, more homogeneous phenotypic subgroups. They compared these fixed (latent) phenotypes with temporal changes of clusters consisting of the association rules. However, the results of this hybrid methodology are challenging for interpretation in the real-life context. Without the knowledgeable domain expert’s input, such procedures might turn into endless and meaningless mining over the data streams.

Our research group has long-term experience in applying ML methods for solving complex clinical tasks associated with chronic aging diseases. Our studies provide a confirmation that in medical research, there are often conditions for small dataset studies. This appears when there is a need to perform research at the institutional level, or when eHRs are not transformed into a modality for research, or when the size of the dataset is constrained by the complexity and high cost of large-scale experiments [70]. The ideal platforms for exploratory studies represent all the properties of the investigated populations. In line with this approach, small studies require that participants be described with multiple features [71,72].

From the perspective of our own experience, we can state that close collaboration and a good mutual understanding between the medical expert and the data analyst is necessary throughout the entire process of data analysis, from data collection (selection), methods selection to the interpretation of the results, and in any case of problem-solving tasks [73]. We can also state with confidence that there is a need for using several analytical methods in most tasks, where data visualization techniques can substantially improve understanding of the results. Yet by combining and integrating all results within the common context, we can achieve a more comprehensive view of the problem of interest [74].

Our work that was focused on discovering the health status components that determine older primary care patients with multimorbidity who responded poorly to influenza vaccination can be considered a pioneer work in approaching the problem of complexity of multimorbidity [75]. By learning through our own work, we have realized that the clinical context’s high dimensionality in older people with multiple comorbidities, due to phenomena such as multiple relationships, non-linearity, and overlapping between disorders, makes the difficult division of individuals into clearly separated groups. In this case, and if the clinical endpoint is a binary label (positive vs. negative disease or procedure outcomes), methods should be used for patient phenotyping that is robust and does not require strong assumptions on the distributions and interdependence of the predictors, such as, i.e., ANNs [76]. The high dimensionality of the data can be reduced by using some of the subspace analysis techniques, such as “subspace clustering”, complemented with the visual exploration methods [77]. The recent developments in constructing different DL methods for feature extraction from eHR data have shown successful inpatient phenotyping and outperforms the classical feature extraction algorithms [78]. When using these techniques, the challenging issue is interpretation of the results, as the analyst may choose between several model options. Different parameter settings may result in different model sets. Besides, various reasons may cause the outcome, and the domain expert should discuss the reliability of the results.

The ML/BD-AI approaches also have disadvantages, which makes the barriers for the direct implementation into clinical practice of the research results obtained by using these approaches (Table 4) [79,80,81,82].

## 4. Current State and Future Perspective in Using Machine Learning/Big Data Analytics in Research on Multimorbidity

### 4.1. New Approaches in Multimorbidity Research Associated with Patterns and Clusters

The ML/BD methods in multimorbidity research have firstly been used in epidemiologic surveys in order to reduce the large number of disease combinations, as obtained by numbering disease dyads and triads, and to try to unify the disease patterns between the studies, by learning on the spontaneous disease gathering [10,83]. Different methods have been used, including logistic regression analysis, hierarchical clustering methods, such as LCA, and exploratory factor analysis, whereas the data sources included eHRs, national registries, and questionnaires filled out by primary care physicians [84,85,86]. Overall, and as the comparative method analysis has recently confirmed, multimorbidity patterns, although some overlap can be observed due to domination of the frequent diseases in the multimorbidity patterns, vary depending on the spectrum of diseases, investigated population, and the method of analysis, indicating the need for study design standardization [87]. Based on the results of this study, it is suggested that the factorization methods are better to use for describing comorbidity relationships, and that clustering methods are more useful as exploratory studies, when performing in a depth analysis. ARM is typically used to investigate disease associations and explore common patterns [88,89,90]. The tree-based approach produces results that allow an identification of specific combinations of chronic conditions or syndromes [91,92].

Our research group used non-hierarchical (k-means) and hierarchical (LCA) cluster analyses in the pilot exploratory study, aimed at getting some insights into the mechanisms that might have stayed behind the clustering of physical frailty and cognitive impairment–the two major aging entropy states [93,94]. We had firstly targeted these functional disorders, and then provided descriptions of the identified clusters by assessing differences in diagnoses of chronic diseases and other clinical and socio-demographic variables, by means of phenotyping the heterogeneous patients at risk for these outcomes. On the contrary, the expert author group from Italy, had performed the disease-based clusters, and then assessed differences in clinical and functional status of individuals in the clusters, such providing some insights into the mechanisms underlying disease clustering [95]. These authors applied the fuzzy c-means algorithm as a clustering method, also known as “the soft clustering method” [96]. This method is more scalable than the classical “hard clustering methods”, as uses the distribution of probabilities, rather than the level of similarity, among the objects (features or individuals), as the basis for assigning a membership to the clusters.

Over the past few years, there is an increasing trend in using methods of DL and data from eHRs for the tasks such as patient phenotyping, disease feature detection or classification, and clinical outcomes prediction based on longitudinal sequences of events, that are expected to improve solving tasks associated with multimorbidity [78,97]. Many developments in DL are used for mapping reliable concepts in raw or minimally-processed data in eHRs (embedding techniques), including textual medical notes, which concepts are then used for temporal sequencing and predicting the outcomes [41,98]. For example, Meng et al. used information on lines of therapy for cancer patients from the large insurance claim dataset to identify treatment pathways. These authors created an algorithm to derive a patient-level lines of therapy and aggregated this information via clustering and data visualization methods, to derive temporal phenotypes and support disease progression prediction [99]. Zhao et al. applied a modified non-negative tensor-factorization approach (a technique used for discovering latent object variables in image analysis) on eHRs data in order to identify phenotypic subtypes in patients at risk for cardiovascular disease. By combining ARM with the estimated risk of each subtype for the development of cardiovascular disease, these authors could identify some previously unknown phenotypes [100]. Nguyen et al. developed a modified convolutional neural network (CNN) model for predicting the probability of hospital readmission, based on medical history information used as a sequence of concepts [101]. Choi et al. developed a modified recurrent neural network (RNN) model for predicting diagnoses and medication prescriptions in the subsequent visits [102]. Although these case studies were aimed at facing the complexity of chronic diseases, they still hardly cope with the complexity of multimorbidity.

Some techniques from the reinforcement learning framework are used to provide to physicians the data-driven decision support for the treatment options that will likely to optimally prevent disease worsening, based on predicting future health states by using longitudinal sequences from eHRs [103].

A general trend, nowadays, in assessing multimorbidity, is to move from disease-only to multi-modal presentation of phenotypes, including also information on medications, laboratory findings and functional health status, in addition to disease labels, in order to achieve better understanding of disease pathways. To meet this challenge, new algorithms and matrices have been developed, with improved capabilities to handle large and multi-modal datasets, and to extract hidden information from them, as presented in the recent review paper of Hassaine et al. [31]. The major innovations include a shift from static to probabilistic implementations of basic ML methods, that allows a shift from qualitative descriptive to quantitative test methods, and developments in “deep phenotyping”. The latter term indicates efforts for establishing features of patient subgroups that are comprehensive enough to be stable across the layers of the complex data structures, and during the phenotype’s progression or the generation of new disease connections [104].

The presentation of temporal dynamics of multiple interactions inherent to multimorbidity is a special challenge for data scientists as it requires more sophisticated algorithms and a multi-step analytics, that go beyond a case-control classification and the use of linear regression analysis to show its progression over time [105,106,107]. The study designs for learning about temporal dynamics of this complexity are still poorly developed and are maintained within the framework of unsupervised learning (an outcome is not known) and disease-disease relationships [31,107]. Although data scientists have an absolute authority in building these sophisticated data analytics, without incorporation of domain knowledge in creation of the research task and evaluation of the interpretability of the performed analytics, the real-life usability of these innovations will be questionable [104]. When considering questions associated with multimorbidity, it may require inference to be made under the conditions of uncertainty and incomplete and contradicting knowledge. Methods different from traditional ML, such as the argumentation theory, can better perform in learning such tasks [108,109]. For these new models, the knowledge of the domain expert might be critical for fulfilling the research task.

### 4.2. The Ways to Improve Implementation of Machine Learning/Big Data Approaches in Research on Multimorbidity

Examples that follow reveal some essential potentials of the ML/BD analytics in the healthcare domain, including the possibility of finding new concepts from routinely collected data to support diagnosis and even improve disease classification, as well as the possibility of using unstructured data, such as the plain text, or images, that otherwise could not be impossible. Automatization, a feature representation without the need for manual efforts and ad hoc input by an expert, is another possibility. Finally, ML/BD analytics is possible in linking eHRs from different platforms and healthcare settings.

Liang et al. constructed a clinical decision support system so that they applied a DL model (a modified version of deep belief network) to extract general concepts from the medical notes (unsupervised task) used from the outpatient clinic and hospital eHRs [110]. The original datasets were combinations of unstructured data (written symptoms and signs in the plain text) and structured data (laboratory data and socio-demographic data). After the network of the DL model had been trained to obtain features from the original (raw) data, the network parameters of the DL model (weights) were tuned in a supervised manner by using SVM as a classical classification model. The extracted features (encoded by the hidden layers of DL model) were further modelled to fit the target outcome measures. Those were diagnoses of some common diseases encoded according to the international classification.

Many similar projects can be found in the recent literature, where authors used either the disease-specific registries or eHRs, and either time-sequential or cross-sectional patterns, to model patient features to support/predict diagnosis disease activity. Norgeot et al. applied a longitudinal deep learning model to model structured data (indicating medications, laboratories, patient demographics and disease activity) from two large hospital registries of patients with rheumatoid arthritis, to predict disease activity at the next visit [111]. By comparing the models’ performances from the two settings, it is possible to assess the quality of care and evaluate the model interoperability. The aggregated eHRs of about 700,000 patient records from the Mount Sinai data warehouse were used to perform unsupervised patient representation models. The models were evaluated by assessing the probability of patients developing certain diseases [112].

The presented examples demonstrate a high potential of the ML/BD research approach in improving diagnosis and prognosis for single chronic diseases, which otherwise may be complicated and labor-intensive procedures. However, the design of these studies still does not capture the full range complexity of multimorbidity, because single diseases are used as target variables and because of the lack of patient division into subgroups that could more specifically reflect differences in developmental disease stages.

The design of the study of Peng et al. is closer to this concept [113]. The authors used the Taiwanese national health insurance research database to develop the ML (a random forest method) cumulative deficit frailty index (a data-driven approach) and compare it to the conventional index for which the selection of features is based on expert opinion (a hypothesis-driven approach) [114]. What is interesting in this study is that authors stratified patients in several groups according to the risk for important adverse outcomes, such as all-cause mortality, hospitalizations, and intensive unit care admissions, using survival analyses (the Kaplan-Meier survival curves and Cox models); classical statistical methods. This study’s design is coherent with the integrated theory of aging, proposing aging as the subsequent decline in functional capabilities of older individuals due to disruption in physiological connections and the dispersion of phenotypes [39,40].

Like the idea of this study, our research group assumes that, when studying multimorbidity problems, the multi-modal data that describe patients with many aspects should create the input of classification or prediction models. That measures of health status functional decline, rather than disease labels, should be used as the outcome measures [93,94]. In line with the paradigm of complex thinking, the authors in this study used a combination of well-proven methods, prioritizing the problem-solving task over the assessment of new techniques.

Managing treatment recommendations for patients with multimorbidity is a difficult problem due to complex disease and medication dependencies. Zhang et al. proposed an algorithm to decompose the treatment recommendation into a sequential decision-making process while automatically determining the appropriate number of medications based on reinforcement learning [115] (RL). It removes 99.8% of adverse drug interactions in the recommended treatment sets. Zheng et al. trained an RL prescription algorithm recommending a treatment regimen optimizing patients’ cumulative health outcomes using their individual characteristics and medical history. In general, the application of RL in healthcare is focused mainly on treatment recommendation problem. However, in practice, it is quite unpractical to identify relevant outcomes for every performed action during the medical process.

Also, challenging, in older persons with multimorbidity, is the problem of predicting medical risks. This question has been traditionally answered by experienced clinicians, who have seen many patients and are familiar with clinical guidelines, or by linear prediction models with well-defined risk factors. Both strategies being more suitable in the context of a single disease than in the context of multimorbidity [6]. Pham et coll. presented an advanced temporal architecture based on using a deep dynamic neural network method for predicting disease progression, recommended interventions, and hospital admissions, based on medical history information [116]. The authors solved important problems in predicting future risks based on temporal sequences used from eHRs, such as long- and short-term dependency between changes in health status, irregular timing and episodic recording of episodes of care, and interactions between interventions and disease progression. This solution is still insufficient to handle the complexity of multimorbidity, as it uses only coded features from eHRs (diagnoses, procedures, and medications) and targets single diseases’ progression. In the study of Hassaine et al., it was shown, using the matrix factorization method, how disease clusters progress over time, forming multimorbidity networks. An interpretation of the results is questionable, as the real clinical utility is not easy to see [107].

The research group from Catalonia has been engaged in defining multimorbidity patterns in a large elderly (≥65 years) population from primary care, using data from Information System for Research in Primary Care (SIDIAP) [117,118,119]. Primary care setting, where older patients with chronic medical conditions regularly encounter and where information on their health history and different aspects of care are routinely collected in eHRs, represents a unique point for longitudinal analysis of multimorbidity patterns. In the recent cross-sectional study (2019) they used a fuzzy cluster analysis (allows individuals to be linked simultaneously to multiple clusters, that is more consistent with clinical experience than other approaches) to identify multimorbidity clusters, and obtained something different clusters than in an older study (2012), where they used a combination of multiple correspondence analysis (for categorical variables) and k-means clustering (for numerical variables) to identify clusters [117,118]. In this older study, the authors followed the obtained clusters for six years and showed that the initially defined clusters are relatively stable on change over time (as determined by the number and % of patients retained in the cluster at the end of the follow-up period).

In their most recent study, this group of authors at baseline used a cross-sectional design and a fuzzy cluster analysis to identify multimorbidity patterns, and then modelled longitudinal trajectories of multimorbidity patterns with a Hidden Markov Model (an approximation for solving complex ML and reinforcement learning problems when, i.e., there is a need for modelling transition across multimorbidity patterns and mortality risk), with some additional algorithms used for linking the inter cluster transition probabilities with the initial cluster probabilities [119]. The authors’ estimated a five-year survival rates for multimorbidity patterns with Cox regression models. Additional variables, indicating socio-demographics and the number of medications and visits, were used to analyse the pathophysiology background of disease clustering and the cluster temporal evolution. The multimorbidity trajectories were shown generally stable over time, which makes a basis for on-time targeting specific multimorbidity patterns with preventive measures.

The Catalonian research group’s presented studies are more clinically sound than most others and give some directions for the population management of multimorbidity. A major complaint is that functional impairments, such as frailty, are not included in the analysis, while it is known that frailty status may significantly change disease presentation and mortality risk [120]. Moreover, information on laboratory tests and body shape measures, as we showed in our work, may provide additional information for disease severity stratification due to the progressive nature of chronic diseases [93,94].

That the number and type of chronic diseases implicate the level of physical and overall functioning over time and with advancing age, was shown in the studies of Stenholm et al. and Vetrano et al. [105,121]. Modelling the temporal transitions of disease clusters with the decline in older persons’ functional capabilities with multimorbidity jointly, is a further challenge in modelling the multimorbidity complexity.

The studies mentioned above have revealed some puzzling situations that burden the actual research on multimorbidity. The problem has its clinical science perspective and the data science perspective. Concerning the clinical side of the problem, a significant concern is the lack of consensus on the definition of multimorbidity and the variable scope of diseases (or other disorders) used to create the clusters [122]. The heterogeneity of cluster identification methods further contributes to the variability of multimorbidity patterns that can be found in various studies [123]. Only a few studies on the temporal evolution of multimorbidity patterns are insufficient to guide future study designs.

On the data science side, the problem lies in the rapid advancement of ML/BD algorithms with ever improving modeling performance, which makes it enormously difficult to compare studies and further delays the implementation of research results [124]. Moreover, variations in datasets, and a range of ML/BD methods that are available for analysis of the same task, may cause variations of the results, and investigators often simultaneously apply several methods to compare their efficiency [125]. Despite the undoubted benefit to research, routinely collected data from eHRs have been established primarily for administrative and clinical trial purposes, making these data potentially inadequate for proposed research [126,127]. Data from eHRs also suffer from shortcomings such as data incompleteness, irregular sampling, and data imbalance, which need alternative methods in the evaluation of machine learning algorithms for medical classification and diagnostic testing [128]. Data scientists are developing various pre-processing and dimensionality reduction methods and approaches to overcome these shortcomings [129]. For solving complex tasks, data scientists use a combination of methods or create complicated protocols, which differ inefficiency to one another. Although there is a tendency for the absolute automatization of the analytic process, which aims to reduce the manual efforts, without the influence of the domain expert in all phases of data analysis, the obtained results may be too complicated, puzzling, and practically useless [97,104].

In trying to solve these problems, consensus groups should be formed among medical experts as well as data scientists and AI experts who, each on his or her own side, would move things forward. By working together in interdisciplinary teams, these expert groups could facilitate the validation and standardization of methods and the establishment of common research protocols, like the process of developing clinical guidelines. The creation of a list of well-defined research questions or target outcomes by medical experts is a prerequisite for identifying patient risk groups, modeling data from electronic health records, and creating the most appropriate care plans. Population health management would be more meaningful than it is today if an efficient and less time-consuming process for assessing risk for older, multimorbid patients in primary care were established and continuously improved. To participate more competently in this process, physicians, especially general practitioners, who are the first to encounter multimorbid patients and have the greatest responsibility for data collection in primary care physicians’ eHRs, should receive more training in the capabilities of ML/BD techniques and quantitative methods for data analysis [130].

## 5. Conclusions

Population-based health management for older people (≥60 years) is inadequate. This is largely because the delivery of personalized care and preventive interventions is limited by the inadequacies of traditional research designs and data analysis methods. This large segment of the population is characterized by increasing complexity of chronic diseases and multimorbidity (two or more chronic diseases in the same person). An alternative research approach based on ML/BD methods has emerged as a real alternative with high potential to address the problems associated with multimorbidity. These problems include, for example, phenotyping of patients and risk stratification based on modeling of multiple interrelated traits that overlap between individuals. Further, identifying patterns of chronic health conditions in the population, as well as tracking progression as health conditions worsen over time in individuals with multiple health conditions. The challenges that need to be addressed to successfully implement this research approach into routine clinical practice mainly relate to the need to establish better coordination between medical experts and data scientists, AI researchers, and IT experts to implement common and validated research protocols tested in real-world conditions and to build a true interdisciplinary knowledge base. From this growing knowledge base, consisting of case studies solving various problems arising from clinical practice, it will be possible in the future to develop new interdisciplinary-based guidelines and recommendations for the management of multimorbidity.

## Figures and Tables

**Table 1:** Descriptions of the key terms in Machine Learning and Big Data AI research approaches [41,42,43,44,45].

Key Term	Description
Knowledge Discovery (KD)	A multiple-step process in data analysis, often managed using CRISP-DM methodology including steps: (1) business understanding; (2) data understanding; (3) data preparation; (4) modelling–decision models generation, patterns extraction; (5) evaluation and (6) deployment-the new knowledge implementation in practice.
Data Mining (DM)	Some experts use it to name the knowledge discovery process. Other experts view data mining as an essential step in the process of knowledge discovery = modelling.
Machine Learning (ML)	The engine within the framework of AI; the collection of techniques allowing computers to undertake complicated tasks by implementation of learning on data (by training and validating the data). The main ML categories are Supervised (SV) Learning, Un-Supervised (USV) Learning and Reinforcement Learning.
The Big Data analytical approach	Enables managing data of the big size and high diversity and complexity; its emergency is due to the rapid advances of high-throughput (-omics) technologies and a wide adoption of eHRs; it is able to challenge the paradigm shift in research on multimorbidity towards the logic of the precision medicine.
Precision medicine	Marked with 4P: Personalized, Predictive, Preventive and Participatory-individualized evaluation and treatments-in contrast to the paradigm “one-size-fits-all”.
The black box concept	Refers to models that use nonlinear transformations to facilitate feature identification; it is used in complex algorithms, such as Artificial Neural Networks (ANN) or a new concept called Deep Learning (DL).

**Table 2:** The list of methods in Machine Learning/Big Data-AI research approach [31,41,42,46].

Method	Description
SV Learning algorithms	A model is trained on a range of input data that are associated with a known outcome (but there is no knowledge on predictors).
USV Learning algorithms	Does not involve the knowledge of the outcome; they are usually used to find undefined patterns or clusters in datasets or to reduce the number of features.
Reinforcement Learning	The algorithms do not need to know the outcome; they use the estimated errors as rewards or penalties.
Association Rule Mining (ARM)	Techniques aim to observe frequently occurring patterns, correlations, or associations in the data; how items are associated to each other.
Classification techniques	The objects are assigned to one of a pre-specified set of classes. Some classification techniques are: Logistic regression (LR) Naive Bayes (NB) methods Decision Trees (DTs) Random Forest (RF) (an adaptation of DTs) ANN Bayesian networks (BNs) Support Vector Machine (SVM)
Clustering techniques	The objects are grouped without any pre-specified knowledge on the rule of their grouping (based on using the distance metrics). Some clustering techniques are: K-means K-Nearest Neighbor (KNN) Principal-component (PC) based clustering Self-organizing maps (SOMs) Latent Class Analysis (LCA)
Deep Learning	More recent concept of ML; has much better ability of feature representation in the abstract level; has an ability to translate the information from the high level of an abstraction to the level that is more understandable for human reasoning; uses complex algorithms, such as ANN.
Advanced computer-based methods	Techniques that can be used to organize highly complex or unstructured data or to find temporal trends in data: Graph-based DM Data Visualization and Visual Analytics Topological DM Fuzzy set theory-based algorithms Natural Language Processing (NLP) methods Dynamic BNs Temporal Association Rules (TARs) Non-negative Matrix factorization (NMF) and tensor factorization (NTF) approaches and their developments in DL

**Table 3:** The arguments to use the Machine Learning/Big Data-AI approaches in research on multimorbidity [29,30,45].

Arguments
Managing data of different grades of diversity and complexity.
Allowing for hidden knowledge to be extracted from data.
The potential to represent real world phenomena.
Linking data of different types and of multiple data sources.
Clinical research tasks determine research methods, which is opposite to what is nowadays when clinical projects meet the criteria of the established research methods.
In predicting the behavior of the system, the method learns from data.
Making sense of all accumulated data (including data from routine medical practice).
Patterns identification or identification of temporal trends in patterns.
The crucial role of a domain expert (knowledge) in data analyzing and in interpreting the results.
Application in different areas of research on multimorbidity, including: Population management and prevention program planning. Health status prediction and prognosis. Drug safety surveillance in the context of polypharmacy and comorbidities. Health risk stratification and personalized treatments. Patient characteristics diversity. Clinical decision support. Prediction of outcomes based on multiple features. Quality of care / performance measurement. Complex problem-solving tasks.

**Table 4:** Disadvantages in using the Machine Learning/Big Data-AI approaches in research on multimorbidity [14,78,79,80,81,82].

Statements
Typically, require massive data samples for training.
High data quality without missing or biased values.
Enough time for model’s generation in combination of training and testing.
Insufficient prediction performance for clinical practice
Results interpretation, transparency and explainability.
More accurate quantitative measures to evaluate the utility and privacy preservation.
Insufficient validation for clinical practice
High error-susceptibility.
